# Stimulation of Innate and Adaptive Immunity by Using Filamentous Bacteriophage fd Targeted to DEC-205

**DOI:** 10.1155/2015/585078

**Published:** 2015-08-26

**Authors:** Luciana D'Apice, Valerio Costa, Rossella Sartorius, Maria Trovato, Marianna Aprile, Piergiuseppe De Berardinis

**Affiliations:** ^1^Institute of Protein Biochemistry (IBP), National Council of Research, 80131 Naples, Italy; ^2^Institute of Genetics and Biophysics “A. Buzzati-Traverso” (IGB), National Council of Research, 80131 Naples, Italy

## Abstract

The filamentous bacteriophage fd, codisplaying antigenic determinants and a single chain antibody fragment directed against the dendritic cell receptor DEC-205, is a promising vaccine candidate for its safety and its ability to elicit innate and adaptive immune response in absence of adjuvants. By using a system vaccinology approach based on RNA-Sequencing (RNA-Seq) analysis, we describe a relevant gene modulation in dendritic cells pulsed with anti-DEC-205 bacteriophages fd. RNA-Seq data analysis indicates that the bacteriophage fd virions are sensed as a pathogen by dendritic cells; they activate the danger receptors that trigger an innate immune response and thus confer a strong adjuvanticity that is needed to obtain a long-lasting adaptive immune response.

## 1. Introduction

Vaccines are one of the most successful outcomes of modern medicine in improving the global health. Nevertheless, many diseases are still a challenge for vaccine development and the attempt to make new vaccines using more recent technologies has required the use of adjuvants, which enhance the magnitude and modulate the quality of the immune response.

In this context, recent failure in producing functional vaccines against emerging diseases has shown that formulating a vaccine able to induce a protective immunity should involve the innate immunity. To this purpose, adjuvants should be natural ligands or synthetic agonists for pattern-recognition receptors (PRRs) that are the molecules responsible of sensing microbes. Among the PRRs, toll-like receptors (TLRs), C-type lectin-like receptors, and the cytosolic NOD-like receptors sense a broad range of microbial* stimuli*, and the cytosolic RIG-I-like receptors sense viral nucleic acids [1].

PRR activation stimulates the production of proinflammatory cytokines/chemokines and type I Interferons (IFNs) that increase the host's ability to eliminate the pathogen. Thus, the incorporation of pathogen associated with molecular patterns (PAMPs) in vaccine formulations can improve and accelerate the induction of vaccine-specific responses.

The adjuvants currently licensed for human use are alum, an aluminum salt-based adjuvant, AS04, an adjuvant composed of monophosphoryl lipid A (MPL) adsorbed to alum, the oil-in-water emulsions, such as MF59 and AS03, and virosomes, composed of lipids and hemagglutinin [2]. Each of these approved adjuvant components has drawbacks: aluminium-based adjuvants determine macrophagic myofasciitis and delayed-type hypersensitivity [3], while both AS04 and MF59 have cost limitations due to the expensive process of MPL purification and the use of nonrenewable resource as shark oil (for MF59). These considerations highlight the need to develop new types of adjuvants able to interact with the innate immune system.

Here we propose the filamentous bacteriophage antigen display system as a candidate vaccine able to induce both the innate and the adaptive response. This system is based on a nonpathogenic procaryotic virus, well characterized at both structural and genetic level [4]. The use of bacteriophage as antigen delivery system is based on modification of the phage display technology. In particular, it is designed to express multiple copies of exogenous peptides (or polypeptides) as fusions to viral capsid proteins. Recombinant virions that carry multiple copies of exogenous sequences can be easily generated cloning a double strand DNA fragment in the phage genome. The protein used to display short antigenic peptides on the phage surface is the pVIII [5]. Such protein, with 2,700 copies per wild type virion, allows the display of a large number of foreign antigenic sequences. Its major limitation relies on the number of amino acids that can be displayed without disrupting the phage assembly [5]. The best strategy to display long exogenous polypeptides is to use the pIII protein, which allows accommodating even a whole protein on the viral surface, although in a maximum of five copies per virion [6].

Thus, the ability of filamentous bacteriophages to tolerate recombinant coat proteins showing short peptides (on pVIII) or bigger polypeptides (on pIII) makes this virus appealing as antigenic carrier. Indeed, it has been already demonstrated to be a powerful delivery system in numerous vaccine development studies [7–11].

We have previously described that the filamentous bacteriophage, when engineered to express antigenic epitopes, elicits T cell help [7] and triggers cytotoxic T cell-mediated response [8]. More recently, we have further improved this delivery system by targeting fd particles to dendritic cells (DCs)* via* DEC-205, an endocytic receptor expressed mainly by dendritic cells [11]. DEC-205 is a C type I lectin-like receptor with ten CRD-like domains and a cytoplasmic tail containing a membrane proximal tyrosine-based region for internalization in clathrin-coated vesicles and a distal region with an EDE amino acid triad for the targeting to late endosome and lysosome and for the recycling to cell surface. DEC-205 is able to internalise and deliver antigens to late endolysosomal compartments allowing the degradation and enhancing efficiency of antigen presentation by dendritic cells [12]. Therefore, it represents a promising receptor for antigen delivery in dendritic cell-targeted vaccines.

**Figure 1:**
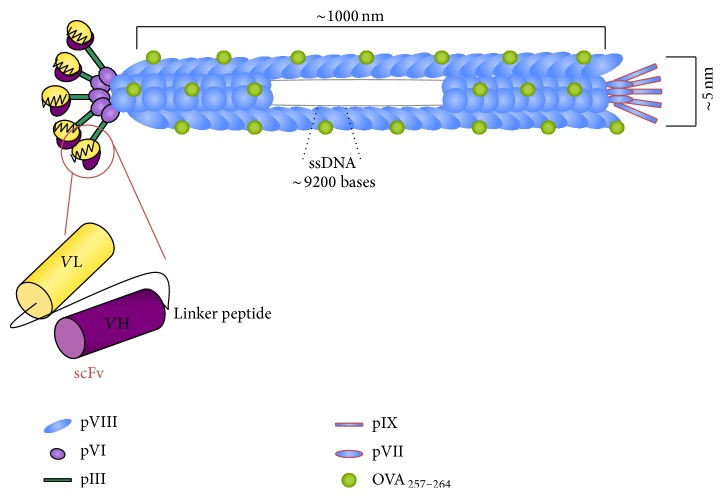
Schematic representation of the engineered filamentous bacteriophage fdOVA/sc-*α*DEC displaying single chain variable fragment (scFv) anti-DEC-205 molecules as fusion to pIII proteins and the OVA_(257–264)_ peptide as N-terminal fusion to pVIII proteins. The scFv is composed of heavy (VH) and light (VL) chain variable regions of the mouse monoclonal antibody NLDC145, assembled with a (Gly4Ser)3 linker to yield a single-chain fragment binding the mouse dendritic cells DEC-205 receptor. About 9200 bases of engineered genome include about 6400 bases of wild type DNA, the *β*-lactamase gene conferring the Ampicillin resistance, one additional copy of pVIII gene plus bases coding for the OVA_(257–264)_ peptide, and the sequence encoding for the single chain antibody fragment anti-DEC-205 plus the HA tag.

As a proof of principle, we have produced a double hybrid bacteriophage expressing the antigenic determinant OVA_(257–264)_ cytotoxic peptide at N-terminus of the pVIII protein and the single chain variable fragment of the NLDC145 antibody directed against the mouse DEC-205 receptor (Figure 1). We have demonstrated that this double-displaying bacteriophage induces stronger antigenic response if compared to nontargeted bacteriophage, enhancing uptake by dendritic cells and inducing DC maturation [11].

The double recombinant bacteriophage represents a powerful delivery system able to target specifically DCs, to promote DC maturation, and to induce specific CD8^+^ T cell response even if administered in the absence of adjuvants or maturation* stimuli*. The ability of fd bacteriophage targeted to DEC-205 (fdsc-*α*DEC) to induce this strong immune response in the absence of exogenous adjuvants is due to DEC-205-mediated delivering of fd particles into endolysosomal LAMP-1+ compartments and their subsequent colocalization with the innate immune Toll like receptor TLR9 [13]. TLR9, which detects CpG-rich viral DNA, is thus activated by the single-strand DNA genome rich in CpG motifs, and this activation leads to an enhanced immunogenicity of the antigenic determinants displayed on the bacteriophage coat [14].

Using a system vaccinology approach based on RNA-Sequencing analysis of bone marrow derived dendritic cells (BMDCs) pulsed with fdsc-*α*DEC, here we report new insights about the molecular mechanisms by which filamentous bacteriophage induces protective immunity. Our data reveal the ability of this valuable antigen delivery system to induce wide changes in the gene expression pattern of dendritic cells. Such modifications mostly overlap with those induced by different pathogens (bacteria, fungi, and protozoan) in the same cells. Many of the differentially expressed genes are under the control of proinflammatory cytokines and in particular of the interferon molecules. Finally, some of the upregulated genes have been recently described and proposed as biomarkers of vaccine efficacy, strengthening the relevance of our findings.

## 2. Materials and Methods

### 2.1. Purification of Bacteriophage Particles and Western Blot

Recombinant fdsc-*α*DEC (expressing a single chain variable fragment against mouse DEC-205 molecule) and fdOVA/sc-*α*DEC bacteriophages (expressing OVA_(257–264)_ epitope and the anti-DEC-205 scFv) were in PBS solution and purified as described previously [11]. The hybrid phage preparations carrying the OVA_(257–264)_ peptide displayed 20% copies of the recombinant pVIII protein, as estimated by N-terminal sequence analysis of the purified virions. Elimination of lipopolysaccharide was performed according to Aida et al. [15] by extraction with Triton X-114 (Sigma-Aldrich, Milan, Italy) and assessed using the limulus amebocyte lysate (LAL) assay (QCL-1000, Lonza, Basel, Switzerland), according to the manufacturer's instructions. The expression of the scFv anti-DEC-205 in the pIII protein of the purified virions was assessed by Western blot analysis using a mouse anti-HA tag mAb (Roche-Boehringer, Basel, Switzerland). Bands of interest were visualized using enhanced chemiluminescence reagent (Thermo Fisher Scientific, IL, USA) and quantified by densitometry (VersaDoc imaging system and Quantity One Analysis Software, Bio-Rad, Milan Italy).

### 2.2. Mice

Six- to eight-week-old female C57BL/6 and ovalbumin (OVA_257–264_) specific TCR transgenic OT-I mice were purchased from Charles River (Lecco, Italy) and were maintained under specific pathogen-free conditions. All experiments with mice were performed in accordance with European union laws and guidelines. All animal studies were approved by our institutional review board and the animal procedures (i.e., immunization and sacrifice) were performed according to rules approved by the ethics committee (permission number 137/2006-a).

### 2.3. Antibodies and Flow Cytometry

Antibodies used for flow cytometry were all from Biolegend and were as follows: anti-CD8-PE-Cy7 (53-6.7), anti-V*α*2-TCR-PE (B20.1), and anti-CD11c-APC (N418). Staining was performed in PBS containing 0.5% BSA for 30 minutes on ice using standard protocols. Data were acquired and analysed by a BD FACSCanto II flow-cytometer and DIVA software (Becton Dickinson, Fullerton, CA).

### 2.4. BMDCs Differentiation and Culture

BMDCs were produced from precursors isolated from the bone marrow of C57BL/6 mice by culturing them with 200 U/mL of recombinant murine granulocyte/macrophage-colony stimulating factor (GM-CSF) (Peprotech, NJ, USA) in RPMI 1640 (Lonza) medium supplemented with 10% FCS, 100 Units/mL penicillin, 100 *μ*g/mL streptomycin, 1 mM sodium pyruvate, and 55 *μ*M 2-mercaptoethanol (all from GIBCO, Milan, Italy). Cells were collected at day 7 of culture and were assayed for their phenotypes of dendritic cells by staining with the monoclonal antibody anti-CD11c.

### 2.5. RNA-Seq Library Production, Sequencing, and Data Analysis

BMDCs were plated in presence or absence of 100 *μ*g/mL of the LPS-purified bacteriophage fdsc-*α*DEC for 20 hours. Total RNA was extracted from cultures using Tri Reagent (Sigma-Aldrich) according to manufacturer's protocol. The integrity and quantity of RNAs were assessed by denaturing agarose gel electrophoresis (presence of sharp 28S and 18S bands) and by spectrophotometry (NanoDrop Technologies). RNA quality was assessed as described in Costa et al. [16]. Paired-end libraries (100 × 2 bp) were prepared using the TruSeq RNA Sample Preparation Kit (Illumina), following the manufacturer's instructions. Libraries were sequenced on the Illumina HiSeq2000 NGS platform at high coverage. A total of about 220 million paired-end reads were sequenced. Reads quality was assessed using FastQC (http://www.bioinformatics.babraham.ac.uk/projects/fastqc/). Mapping to the reference mouse genome (mm9) and to RefSeq transcripts was achieved using TopHat version 2.0.10 [17]. Uniquely mapped reads (about 95% of sequenced reads) were used for further analyses. SamTools and BEDTools were used to convert alignment formats and to produce coverage files (bedgraph format). UCSC Genome Browser was used for quality assessment of mapped reads and to inspect gene-specific features. Cufflinks and Cuffdiff were used to quantify gene expression and to identify differentially expressed genes (DEGs) [18]. PANTHER [19] was used to classify DEGs and DAVID [20, 21], to assess gene ontology, and to perform pathway analysis enrichment on the list of DEGs. Interferon-regulated gene analysis was performed using the INTERFEROME 2.0 bioinformatic database [22].

### 2.6. Adoptive Transfer and T Cell Assays

CD8^+^  OVA_(257–264)_ specific T cells were purified from spleen of OT-I mice using the CD8^+^ T cell isolation kit (Miltenyi Biotec). Cells were labelled by incubation with 1 *μ*M CFSE (Biolegend) for 10 minutes at 37°C. The staining was quenched adding ice cold RPMI1640 media containing 10% FCS.

3.5 × 10^6^ CFSE labeled OT-I CD8^+^ T cells were then injected intravenously into C57BL/6 recipient mice (*n* = 5/group). 24 hours later, mice were immunised subcutaneously with 50 *μ*g of fdOVA/sc-*α*DEC phage particles in PBS 1X. As control, mice were injected with vehicle alone. After 3 days, splenocytes were isolated and stained with anti-V*α*2-PE and anti-CD8-PE-Cy7 mAbs. The CFSE fluorescence intensity of OT-I cells was then evaluated by flow cytometry as previously described [11].

### 2.7. Real-Time Quantitative PCR

Total RNA was isolated using Tri Reagent (Sigma-Aldrich) according to the manufacturer's instructions. For each sample, 500 ng of total RNA was reverse-transcribed into complementary DNA (cDNA) using the High-Capacity cDNA Reverse Transcription Kit (Life Technologies) according to manufacturer's protocol. cDNAs were then used as template for quantitative real-time polymerase chain reaction assays. Amplification reaction mix contained 1x iTaq Universal SYBR Green Supermix (Bio-Rad), 400 nM of each primer, and 25 ng of cDNA (RNA equivalent) as template. PCR conditions were 95°C 30 sec followed by 40 cycles of 95°C × 5 sec and 60°C × 30 sec. Melting curves were generated after amplification using instrument default setting. Data were collected using the CFX Connect real-time PCR detection system (Bio-Rad); each reaction was performed in duplicate. The relative gene expression was calculated using the 2^−ΔΔ*Ct*^ method, and* Actb* was used as housekeeping gene. Primers were designed using Oligo 4.0-s. Sequences of the primers are

  
*Isg15.F*: 5′AGCAAGCAGCCAGAAGCAGA3′,
  
*Isg15.R*: 5′CCCCTTTCGTTCCTCACCA3′,
  
*Irf7.F*: 5′TGCTGTTTGGAGACTGGCTAT3′,
  
*Irf7.R*: 5′GGCTCACTTCTTCCCTATTTT3′,
  
*Il1b.F*: 5′ACAAGGAGAACCAAGCAACGA3′,
  
*Il1b.R*: 5′TGTCCTGACCACTGTTGTTTC3′,
  
*Actb.F*: 5′TTCTTTGCAGCTCCTTCGTT3′,
  
*Actb.R*: 5′GCACATGCCGGAGCCGTT3′.

### 2.8. Statistical Analysis

Results are expressed as the mean ± SD. The statistical significance of differences between experimental groups was calculated using the unpaired two-tailed Student's *t*-test. Results with a *p* value < 0.05 were considered significant.

## 3. Results

### 3.1. *In Vivo* Antigen Specific CD8 T Cell Proliferation after fdOVA/sc-*α*DEC Bacteriophage Immunization

The ability of bacteriophage particles to induce a strong immune response was assayed using the double hybrid bacteriophage coexpressing the OVA_(257–264)_ antigenic peptide and anti-DEC-205 scFv* in vivo* in a mouse model. We inoculated subcutaneously the recombinant fdOVA/sc-*α*DEC bacteriophage particles into C57BL/6 mice that had been adoptively transferred with purified, CFSE-labeled, CD8^+^ T cells derived from OVA_(257–264)_ specific OT-I transgenic mice 24 hours before the immunization. We measured the ability of fdsc–*α*DEC bacteriophage carrying OVA_(257–264)_ to induce an antigen specific immune response, 96 hour after the immunization, by measuring the OVA_(257–264)_ specific CD8^+^ T cell proliferation as CFSE reduced fluorescence by FACS analysis on CD8^+^ V*α*2^+^ gated cells. A group of mice immunized with PBS alone was used as control.

**Figure 2:**
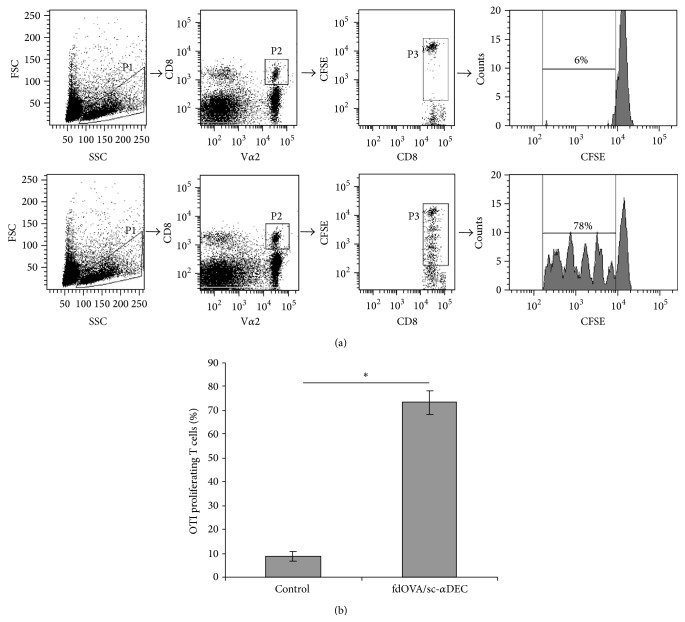
OVA specific OT-I CD8 T cell proliferation. (a) Flow cytometry strategy analysis of OT-I T cells proliferation. One representative sample per group is reported. P1: live cells, P2: OT-I CD8+ V*α*2+ T cells, and P3: OT-I CD8+ V*α*2+ proliferating cells with CFSE fluorescence intensity <10^4^ and >10^2^. The CFSE fluorescence intensity of the P3 population is reported in the histograms and numbers represent the percentage of proliferating cells. Peaks represent the cell division. (b) Mean ± SD percentage of proliferating T cells from adoptively transferred mice (*n* = 5) immunised with PBS or fdOVA/sc-*α*DEC bacteriophage is shown. ^*∗*^ *p* < 0.01.

As reported in Figure 2 the fdOVA/sc-*α*DEC induced a strong proliferative response of the OVA-specific CD8^+^ T cells, in absence of exogenous adjuvant.

### 3.2. RNA-Seq Analysis of BMDC Treated with Bacteriophage fdsc-*α*DEC 

**Figure 3:**
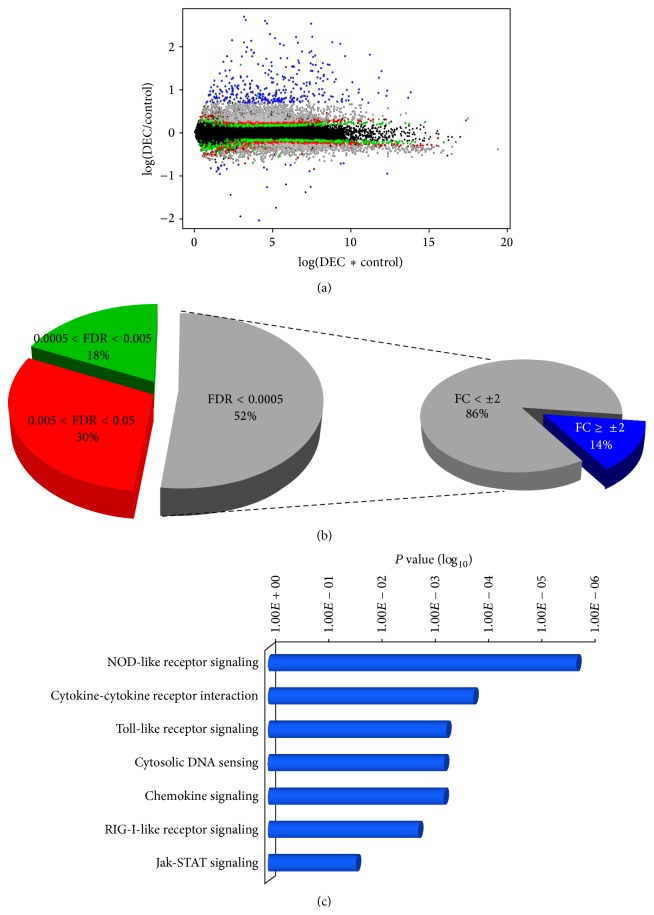
RNA-Seq data analysis of BMDC in presence and absence of fdsc-*α*DEC. MA plot of expressed genes (a) and pie chart (b) of the differentially expressed genes classified according to their FDR value. Genes with FDR < 0.0005 and fold change >±2 are shown in blue. For these genes, pathway analysis is reported in (c).

To gain insights into the molecular mechanisms through which fdsc–*α*DEC induces a strong cell-mediated immune response, we used RNA-Sequencing to analyze the transcriptional profiles of BMDCs* in vitro* challenged with fdsc–*α*DEC phage particles. The gene expression pattern of these cells was compared to the one of control untreated BMDCs (i.e., cells treated only with PBS). Two technical replicates for each condition were performed. Approximately 55 million reads (95% of them uniquely mapped on the reference genome) per replicate were produced. Expression values for both control and fdsc-*α*DEC-treated DCs were measured as FPKM (fragments per kilobase of transcript per million mapped reads). Technical replicates revealed a very highly correlation. Using RNA-Sequencing we could simultaneously measure gene expression levels of (virtually) all genes expressed in mouse DCs. Setting an arbitrary threshold (FPKM = 1) for gene expression, we found about ten thousand genes expressed at significant levels in both conditions. Then, we compared gene expression levels between the two conditions. This analysis revealed that approximately 3800 genes (FDR < 0.01) were differentially expressed (DE) in DCs after exposure to fdsc-*α*DEC compared to control cells (Figure 3(a)). As reported in Figures 3(a) and 3(b), we selected different FDR intervals. Genes with a FDR value between 0.05 and 0.005 are classified in the first group and represent the 30% of the DE genes (in red in Figures 3(a) and 3(b)); the second group includes the 18% of the total DE genes with FDR between 0.005 and 0.0005 (in green in Figures 3(a) and 3(b)), while the most significant DE genes, with FDR under 0.0005, represent the 52% of the total DE genes (in grey in Figures 3(a) and 3(b)). Among them, we further selected DE genes with a fold change (FC) >±2 in DCs exposed to fdsc-*α*DEC* versus* control cells. All further analyses were performed on this group of DE genes named DEG (differentially expressed Genes, shown in blue in Figures 3(a) and 3(b)). Most of these DEG were significantly upregulated in DCs upon treatment with the fdsc-*α*DEC, whereas only very few of them were downmodulated. To understand if these genes with a significant upregulation were related to specific cells function and/or pathways, we interrogated the Database for Annotation, Visualization and Integrated Discovery (DAVID). The most enriched biological pathways (using KEGG database) are shown in Figure 3(c). Interestingly, the exposure of DCs to fdsc-*α*DEC significantly upregulated many genes involved in inflammatory pathways linked to innate immunity.

The pathways with the larger number of upregulated genes were the “NOD-like receptor signaling,” the “cytokine-cytokine receptor interaction,” the “toll-like receptors,” the “cytosolic DNA sensing,” the “chemokine signaling,” and the “RIG-I-like receptor signaling” pathways (Figure 3(c)).

**Figure 4:**
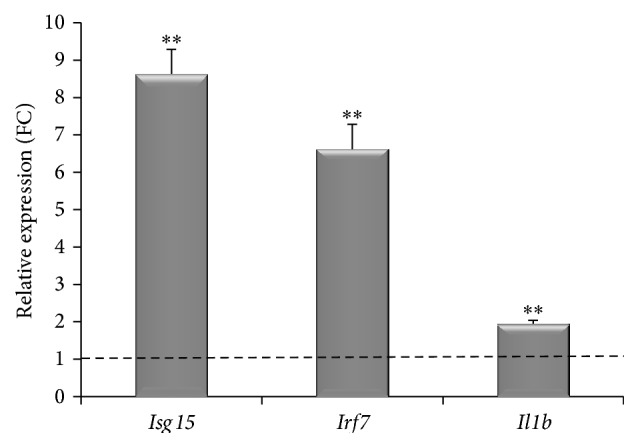
Real-time PCR validation of RNA seq analysis. The* Isg15*,* Irf7,* and* Il1b* gene expression in BMDCs* in vitro* challenged with fdsc-*α*DEC phage particles was measured by real-time PCR. The dashed line corresponds to the mean value of gene expression of PBS treated DCs. Graph shows the fold change (FC) (mean ± SD). ^*∗∗*^ *p* < 0.05.

To better dissect our results, we compared them with the already published data [23] describing mouse DCs treated with phylogenetically different organisms, such as bacteria, helminths, and parasites as a paradigm of how DCs undergo marked reprogramming during infection with live pathogens. Our RNAseq data show that in agreement with data obtained with the live pathogens, our procaryotic virus is able to activate specific classes of genes such as the CXCL1 (growth-related oncogene 1 (GRO1)), CXCL2 (GRO2), CCL2, CCL7, and genes encoding the proinflammatory mediators tumor necrosis factor *α* (TNF-*α*) and interleukin (IL-1 *β*) (see Table 1). Moreover, we also found the significant upregulation (more than 19-fold) of CXCL10 (IFN-inducible protein 10); importantly, this chemokine is essential for the generation of protective CD8^+^ T cell responses and it is produced by dendritic cells following CpG-ODN stimulation [24]. Measuring the expression levels of the antiviral genes Oas3, Oas2, and Eif2ak2 we found that they were 18-, 16-, and 13-fold change increased, respectively, in bacteriophage-treated DCs (Table 1). Finally, expression data revealed a significant upregulation of Interferon-Stimulated Genes (ISG) and in particular of* Isg15* gene that was upregulated more than twentyfold, similarly to* Irf7* gene (Table 1). The expression of these genes was assessed also by quantitative real-time PCR showing a fold change of 8.6 for* Isg15* and 6.6 for* Irf7* gene in DC treated with the engineered bacteriophage (Figure 4). Also the* Il1b* gene expression was measured by real-time PCR and showed a twofold increase of mRNA in fdsc-*α*DEC treated DC.

### 3.3. Interferon Signature on Differentially Expressed Genes

**Figure 5:**
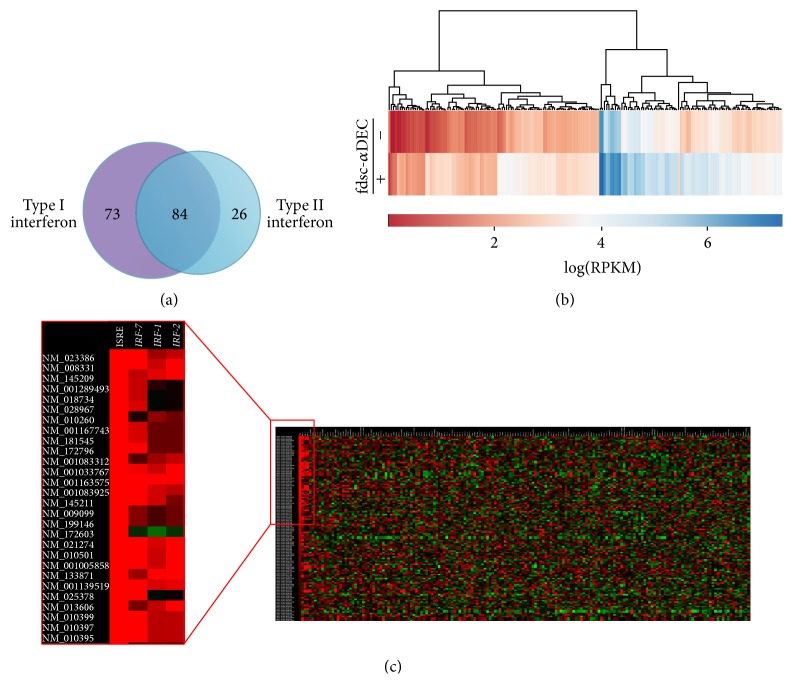
Interferon signature in genes modulated by fdsc-*α*DEC. (a) Venn diagram of the genes analyzed by INTERFEROME 2.0 database. (b) Heatmap of 183 “interferon-related” genes modulated upon fdsc-*α*DEC treatment. Expression values are indicated as logRPKM (reads per kilobase of transcript per million of mapped reads). (c) Heatmap obtained using the online Pscan tool indicating the presence (in red) of transcription factors binding sites (TFBS) within promoters of a selected gene list. In the red box on the left, a detail of such computational analysis is shown.

The genes whose expression was significantly modulated after fdsc–*α*DEC treatment in dendritic cells were analyzed using the INTERFEROME 2.0 database. The analysis revealed the presence of a clear transcriptional interferon signature. Notably, 183 genes out of 361 significantly (FDR < 0.0005) DEG (about 50%) are modulated by Interferon Regulated Factors (IRFs) or by the NF-kB transcription factors and possess Interferon-Stimulated Response Elements (ISREs). The Venn diagram in Figure 5(a) illustrates how many DE genes are regulated* per* interferon type. In detail, 73 out of 183 genes are IFN Type-I dependent, 26 are type-II dependent, and 84 are regulated by both interferons. Expression levels of interferon-regulated genes in DCs, in presence or absence of fdsc-*α*DEC, are shown in the heatmap of Figure 5(b). In addition, we decided to assess whether genes that are affected by fdsc-*α*DEC treatment in DCs have a specific transcription factor binding sites (TFBS) signature within their promoters. To this aim, using Pscan we found that most of upregulated genes have ISREs and binding sites for IRF1, IRF2, and IRF7 molecules (Figure 5(c)). Interestingly, among them are included genes coding for cytokines and chemokines, transcriptional regulators, DNA binding proteins, proteins involved in ISGylation and ubiquitination, and proteins with a known activity in the innate anti-viral response and immune activation (details in Table 1).

## 4. Discussion

It is a current opinion that vaccines should activate the innate immune system in order to start a rapid response to pathogens and sustain at the same time the development of the adaptive immune response. Thus, in order to choose the best vaccine formulations able to stimulate both innate and adaptive immune response, one of the more recent approaches is to take advantage from the analysis of gene expression using the efficient high throughput whole-genome screening. Moreover, using this approach, it is possible to have an overview of how the immune system attacks invading microorganisms, maintains tolerance, and creates a memory of past infections. Up to a decade ago this analysis was based on microarrays, but the development of RNA-Seq methodologies has opened a new era of investigations to identify genes that are differently expressed when samples are treated with different compounds. Moreover, this discovery-based research provides the opportunity to characterize both new genes with unknown functions and genes not previously known to be involved in a particular biological process [25].

In this paper we take advantage from the RNA-Seq with its massive data output in order to elucidate the mechanism by which filamentous bacteriophage antigen delivery system targeting dendritic cells* via* the DEC-205 receptor is able to induce a strong and sustained antigen specific immune response as previously described and represented in Figure 2 [11, 13]. The importance of dendritic cells in initiating immune responses was the key reason for us to select this cell type as target and to investigate at a genetic level how DCs sense this procaryotic virus. DCs reside in an immature state in most tissues, where they recognize and phagocytose pathogens and other antigens. Direct contact with many pathogens leads to the maturation of DCs, which is characterized by an increase in antigen presentation, expression of costimulatory molecules, and subsequent stimulation of naive T cells in lymphoid organs [26].

As a very powerful research tool, the RNA-Seq method shows as a drawback the production of impressive amount of data, and it is up to the researchers to select among them only the more reliable. To be more confident in our study, we analysed only genes with a FDR < 0.0005 and with a fold change in expression level >2; using these genes we started to dissect the gene expression changes in dendritic cells exposed to our vaccine candidate.

Following analysis using DAVID bioinformatics resources, we were able to classify DE genes in pathway categories and we found that the more transcribed genes, after DC challenge, were the ones involved in pathogen recognition for innate immunity activation: genes from the NOD-like receptor and toll-like receptor signaling pathway were upregulated, and the evocated innate immune response started the production of cytokines and chemokines and the up regulation of their receptors. In particular, we found that NOD1 and NLRP3 are upregulated more than 7-fold in DC treated with anti-DEC-205 bacteriophage fd. NOD1 is one of the nucleotide-binding oligomerization domain-like receptors (NLRs), a family of intracellular receptors that detects PAMPs and endogenous molecules; at the same family belongs NLRP3 that is involved in the formation of multiprotein complexes termed inflammasomes that mediates the caspase-1-dependent conversion of pro-IL-1*β* and pro-IL-18 to IL-1*β* and IL-18 [27].

Toll-like receptors belong to the family of pathogen recognition receptors that are triggered by PAMPs expressed by bacteria, viruses, fungi, and protozoa and their stimulation contributes to the induction and maintenance of innate and adaptive immune pathways as well as memory function. In our analysis we registered the upregulation of genes encoding the endosomal toll-like receptors TRL3, TLR7, TLR8, and TLR9.

We previously described that DCs pulsed with bacteriophage fd* via* anti-DEC-205 are able to produce type I IFN [11, 13]; now we found by RNA-Seq analysis that 50% of the reported DE genes are regulated by Interferon Regulated Factors (IRF) and possess Interferon-Stimulated Response Element (ISRE) as reported in Figure 5(c). However, we were not able to detect the upregulation of interferon genes by RNA-Seq analysis, due to the gene organization (only one exon) and high conservation among sequences of the different genes encoding Interferon type I. Since the RNA-Seq method is unable to match the reads on the reference genome, in order to better identify the genes induced by interferon molecules, we used the INTERFEROME bioinformatics tool. From this analysis the interferon signature is evident (see the heatmap in Figure 5(c)), and it is also clear that either type I or type II is involved (see Venn diagram in Figure 5(a)). The type I interferons signal through the interaction with the interferon alpha receptor (IFNAR). Interaction between IFN-I and IFNAR triggers signaling cascades which culminate in the transcriptional regulation of hundreds of IFN-stimulated genes (ISG). One of the first pathways activated* via* IFN-I is the JAK-STAT pathway. STAT1 and STAT2 form dimers and enter into the nucleus to form ISGF3 transcription factor complex, which binds to IFN-stimulated response elements, driving the expression of Isg genes. In our RNA-Seq analysis in BMDC treated with anti-DEC-205 phage particles, we found upregulation of both* Stat1* and* Stat2* (10- and 13-fold, resp., by fd exposure) as well as other genes like* Irf7, Isg15* (confirmed also by real-time PCR, see Figure 4), and* Rig-1* genes, all of which contribute to the positive feedback regulation of the IFN pathway. Moreover, we found upregulation of transcripts of many interferon inducible and anti-viral genes like* Oas, Eif2ak2, Mx1, and Mx2*.

By comparing our results with the already published data on the same subject, in particular in paper describing how host cells undergo marked reprogramming of their transcriptome during infection with live pathogens, we observed a common core of the host DCs-transcriptional program.

In agreement with these data [23], we also observed the upregulated expression (more than 20-fold) of the* Igs15* gene. The function of Isg15 has been recently elucidated: it is ubiquitin-like protein and its activity, named ISGylation, is a modification of target protein and after modification these proteins are conjugated to Isg15. The conjugation of ISG15 to target proteins is reversible and is mediated by conjugating/deconjugating enzymes. ISG15 is conjugated to lysines on numerous target proteins through the action of specific IFN-inducible E1/E2/E3 enzymes. We show here that bacteriophage fd particle uptaken by dendritic cells induced gene expression of* Isg15* and both its conjugating and deconjugating enzymes. In fact, beyond the upregulation of* Isg15*, we found increased levels of transcripts of other factors that regulate ISG15-mediated ISGylation like HECT domain and RCC1-like domain containing protein 6 (HerC6) that represents the main ISG15 E3 ligase, essential for global ISG15 conjugation in mice [28]. Currently, the number of identified ISGylated proteins is increasing, and although the functional role of protein ISGylation is not yet completely understood, there are increasing bodies of evidence to suggest a role in mediating an innate antiviral response. It has been recently demonstrated [29, 30] that the key regulators of signal transduction, such as phospholipase C*γ*1, Jak1, and ERK1, are modified by ISG15, as the transcription factor STAT1, an immediate substrate of Jak1 kinase.

In summary, all our findings indicate the ability of bacteriophage fd to upregulate* via* DEC-205 in the targeted DCs several genes involved in triggering an innate immune response. These findings are in agreement with the already published data on the same subject, in particular in papers describing how dendritic cells undergo marked reprogramming of their transcriptome during infection with live pathogens [23].

## 5. Conclusions

Overall, filamentous bacteriophage fd expressing antigenic epitopes and modified to target the DEC-205 dendritic cell receptor is a valuable vaccine candidate able to induce dendritic cells activation and to trigger antigen specific T cell response.

Here we provide the evidence that bacteriophage fdsc-*α*DEC particles can activate antiviral innate immune responses* via* induction of type I interferons, which in turn orchestrate activation of dendritic cells, enhancing their antigen presenting functions.

From these results we can conclude that DEC-205 fd-targeted bacteriophages have* per se* adjuvant properties and that the fd antigen delivery system combines the safety and capability to trigger a strong cellular antigen-specific immune response even without administering exogenous adjuvants.

## Figures and Tables

**Table 1:** Selection of most significant IFN-related upregulated genes in mouse BMDCs treated with fdsc-*α*DEC bacteriophage vaccine.

Official gene symbol	Gene ID	Description
Chemokine and cytokine		
Il1b	NM_008361	Interleukin-1 beta
Ccl12	NM_011331	Chemokine (C–C motif) ligand 12
Ccl2	NM_011333	Chemokine (C–C motif) ligand 2
Ccl7	NM_013654	Chemokine (C–C motif) ligand 7
Cxcl1	NM_008176	Chemokine (C–X–C motif) ligand 1
Cxcl10	NM_021274	Chemokine (C–X–C motif) ligand 10
Ccr2	NM_009915	Chemokine (C–C motif) receptor 2
Ccr5	NM_009917	Chemokine (C–C motif) receptor 5
Transcriptional factors		
Irf7	NM_016850	Interferon regulatory factor 7
Batf2	NM_028967	Basic leucine zipper transcription factor, ATF-like 2
Znfx1	NM_001033196	Zinc finger, NFX1-type containing 1
Zbtb38	NM_001033196	Zinc finger And BTB domain containing 38
Stat1	NM_009283	Signal transducer and activator of transcription 1
Stat2	NM_019963	Signal transducer and activator of transcription 2
ISGilation and ubiquitination		
Isg15	NM_015783	Interferon-stimulated exonuclease gene 15 kDa
Isg20	NM_020583	Interferon-stimulated exonuclease gene 20 kDa
Usp18	NM_011909	Ubiquitin specific peptidase 18
Usp25	NM_013918	Ubiquitin specific peptidase 25
Herc6	NM_025992	HECT domain and RLD 6
March1	NM_175188	Membrane-associated ring finger (C3HC4) 1
Nucleic acid binding proteins		
Ddx58	NM_172689	Dead box polypeptide 58/RIG-1
Ddx60	NM_001081215	DEAD (Asp-Glu-Ala-Asp) box polypeptide 60
Zbp1	NM_021394	Z-DNA binding protein 1
Eif2ak2	NM_011163	Eukaryotic translation initiation factor 2-alpha kinase 2/PKR
Antiviral proteins		
Gbp2	NM_010260	Guanylate binding protein 2
Gbp3	NM_018734	Guanylate binding protein 3
Gbp4	NM_008620	Guanylate binding protein 4
Oas2	NM_145227	2′-5′-Oligoadenylate synthetase 2
Oas3	NM_145226	2′-5′-Oligoadenylate synthetase 3
Oasl1	NM_145209	2′-5′-Oligoadenylate synthetase-like 1
Oasl2	NM_011854	2′-5′-Oligoadenylate synthetase-like 2
Mx1	NM_010846	Myxovirus (influenza virus) resistance 1
Mx2	NM_013606	Myxovirus (influenza virus) resistance 2
Rsad2	NM_021384	Radical S-adenosyl methionine domain containing 2
